# Endoscopic suturing system is the last chance for rectal bleeding after complicated endoscopic submucosal dissection

**DOI:** 10.1055/a-2342-0164

**Published:** 2024-07-15

**Authors:** Martina De Siena, Federico Barbaro, Vincenzo Bove, Maria Valeria Matteo, Valerio Pontecorvi, Ivo Boškoski, Cristiano Spada

**Affiliations:** 1 18654 Digestive Endoscopy Unit, Fondazione Policlinico Universitario Agostino Gemelli IRCCS, Rome, Italy

We report the case of a 54-year-old woman who underwent endoscopic submucosal dissection (ESD) for a 3-cm laterally spreading tumor (LST) of the mid-proximal rectum ( Fig. 1 ). The ESD was complicated by intraoperative bleeding from visible vessels of the defect. Use of hemostatic forceps (Coagrasper) and endoclip placement led to resolution of the bleeding ( Fig. 2 ).

**Fig. 1:**
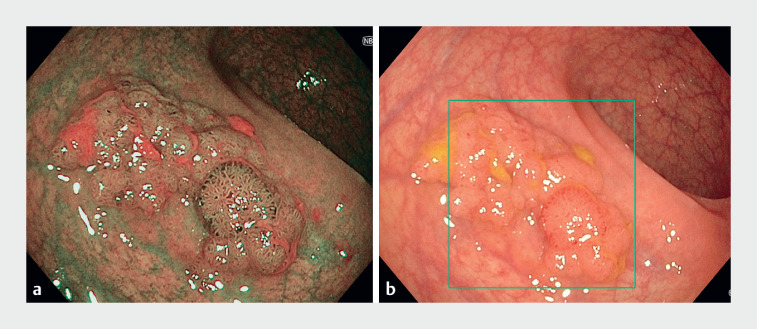
Endoscopic images showing a laterally spreading tumor in the mid-proximal rectum: **a** during computer-aided detection; **b** on narrow-band imaging.

**Fig. 2:**
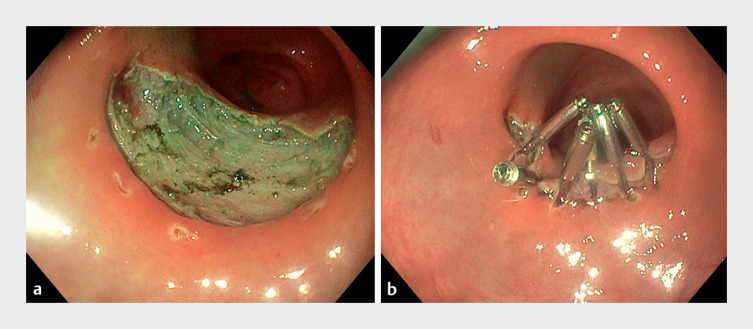
Endoscopic images showing: **a** the post-endoscopic submucosal dissection defect after treatment with hemostatic forceps; **b** the closed defect after the placement of six endoclips.

The patient was closely monitored and no further complications were observed until the second postoperative day when the patient resumed her warfarin therapy because of atrial fibrillation and a mechanical valve prosthesis. The need to resume anticoagulant therapy in patients who have undergone invasive procedures has always been challenging in endoscopy as it can increase the risk of bleeding 1 . In this case, our patient, after a few days of therapy, again developed rectal bleeding. Endoscopic hemostasis was attempted with the injection of fibrin glue and the application of hemostatic powder spray (Hemospray); however, after a period of apparent clinical stability, another emergency endoscopy revealed copious bleeding from the ESD defect ( Video 1 ). This time, after cleansing the area, we removed the previously placed endoclips to better identify the site of bleeding. We then performed endoscopic suturing with a full-thickness suturing device (Apollo Overstitch system), achieving satisfactory control of the bleeding after just one full-thickness bite. Defect closure was refined by the placement of further full-thickness sutures ( Fig. 3 ).

Endoscopic hemostasis is achieved with the use of an endoscopic full-thickness suturing system for recurrent rectal bleeding after endoscopic submucosal dissection.Video 1

**Fig. 3:**
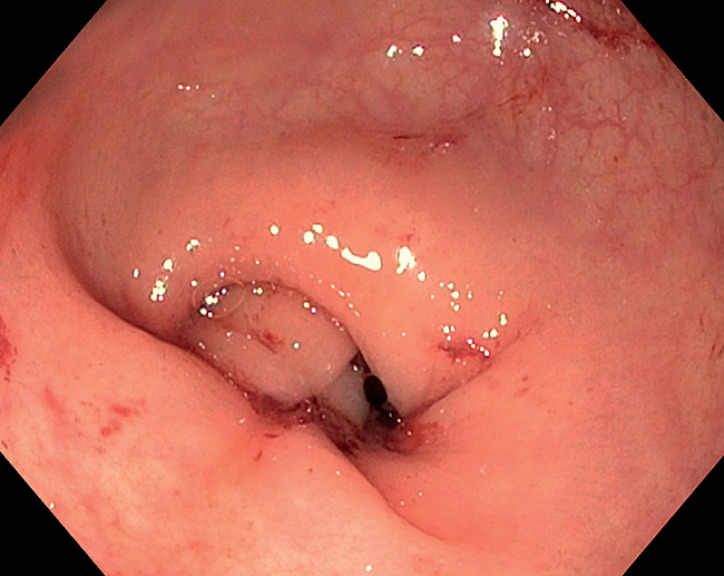
Endoscopic image showing complete control of the bleeding after endoscopic suturing with the full-thickness endoscopic suture system.

After adequate observation, the patient was discharged in good general condition from our hospital and no further bleeding was observed in the following days. Endoscopic follow-up after 3 months showed a flat, regular scar in the mid-proximal rectum, with no signs of recurrence. No rectal stenosis or substenosis was identified after the full-thickness endoscopic suturing.

Several studies have investigated the effectiveness of endoscopic suturing to prevent adverse events after ESD 2 3 . We believe that use of the full-thickness endoscopic suturing device is safe and effective for control of bleeding when other hemostatic methods have failed.

Endoscopy_UCTN_Code_CPL_1AJ_2AZ

## Notes

Endoscopy E-Videos https://eref.thieme.de/e-videos
*E-Videos* is an open access online section of the journal *Endoscopy* , reporting on interesting cases and new techniques in gastroenterological endoscopy. All papers include a high-quality video and are published with a Creative Commons CC-BY license. Endoscopy E-Videos qualify for HINARI discounts and waivers and eligibility is automatically checked during the submission process. We grant 100% waivers to articles whose corresponding authors are based in Group A countries and 50% waivers to those who are based in Group B countries as classified by Research4Life (see: https://www.research4life.org/access/eligibility/ ). This section has its own submission website at https://mc.manuscriptcentral.com/e-videos .
